# Effects of *Cd8a* Gene Knockout on Gut Microbiota and Liver Metabolism

**DOI:** 10.1002/iid3.70557

**Published:** 2026-10-15

**Authors:** Zhongshi Dai, Chuan Wang, Hu Mu, Chunxiu Hou, Jing Tang, Jinhai Ma, Mingxing Zhu, Bingshuo Qian

**Affiliations:** ^1^ School of Basic Medical Sciences Ningxia Medical University Yinchuan Ningxia China; ^2^ Key Laboratory of Common Infectious Disease Prevention and Control in Ningxia Yinchuan Ningxia China; ^3^ School of Laboratory Medicine Ningxia Medical University Yinchuan Ningxia China; ^4^ General Hospital of Ningxia Medical University Yinchuan Ningxia China

**Keywords:** CD8^+^ T cells, gut microbes, liver metabolism

## Abstract

**Objective:**

Evaluate the effects of CD8^+^ T cell defects caused by *Cd8a* gene knockout on gut microbiota and hepatic metabolism, and preliminarily explore the underlying mechanisms.

**Methods:**

The *Cd8a* gene was knocked out in mice to generate *Cd8a* knockout mice. Genotypes were identified using polymerase chain reaction (PCR) and agarose gel electrophoresis. Mice were divided into three groups: normal control, CD8^+^ T cell‐deficient, and CD8^+^ T cell reinfusion, with six mice per group. CD8^+^ T cells from normal control mice were isolated via magnetic bead sorting and reinfused into CD8^+^ T cell‐deficient mice via tail vein injection. Reinfusions were performed four times at 3‐day intervals. Four weeks after the final reinfusion, fecal samples were collected, and DNA extracted for 16S rRNA gene sequencing analysis. Mouse livers were collected and analyzed using non‐targeted metabolomics via liquid chromatography‐tandem mass spectrometry (LC‐MS/MS).

**Results:**

We found that CD8^+^ T cell deficiency‐induced alterations in gut microbiota primarily affected the phyla *Firmicutes*, *Bacteroidetes*, *Proteobacteria*, and *Actinobacteriota*. Following CD8^+^ T cell depletion, the abundance of bacteria such as *Catabacter*, *Vicinamibacter*, *Mesorhizobium*, *Catellatospora*, and *Stomatobaculum* increased (*p* < 0.05); after CD8^+^ T cell reinfusion, the abundance of these bacteria showed a decreasing trend (*p* < 0.05). We also identified five major metabolic pathways in mouse liver that showed significant correlations with CD8^+^ T cell status.

**Conclusion:**

CD8^+^ T cells exert a significant influence on hepatic metabolic responses and gut microbial community diversity.

## 1 Introduction

Throughout infection, T cells experience differentiation into various effector and memory T cells. Particularly, CD8^+^ T cells play a crucial role in adaptive immune responses, essential for cellular immunity, and vital to the immune system's defense mechanisms [1, 2, 3]. CD8^+^ T cells assist in eliminating pathogens by directly targeting cells through two pathways: releasing perforin, granzyme, and increasing Fas ligands. The memory CD8^+^ T cell reservoir allows rapid immune responses upon reencountering the same pathogen, ensuring prolonged protection [4]. After initial activation and expansion, naive CD8^+^ T cells transform into effector CD8^+^ T cells, crucial for eliminating intracellular infections and cancerous cells, playing a significant part in cellular immunity and the body's immune system [5]. The intestinal mucosa, as the body's main defense, contains various unique epithelial and endothelial cells for protection, along with numerous immune cells that operate through innate and adaptive immune responses. Among these, CD8^+^ T cells contribute to maintaining the intestinal barrier by secreting diverse factors and exerting cytotoxic effects.

In specific scenarios such as severe combined immunodeficiency disease (SCID), chronic viral infections like HBV, HCV, and HIV, and following radiotherapy and chemotherapy, T cell levels in the human body decrease significantly [1, 6, 7, 8, 9, 10, 11]. The liver, the largest digestive organ in humans, secretes bile acids into the intestines through the biliary tract, influencing the intestinal microbiota. This process promotes the growth of beneficial bacteria while inhibiting harmful bacteria [12]. The small intestine, essential for digestion and absorption, is densely populated with capillaries, lymphatic vessels, and microbial communities. These characteristics require a substantial presence of immune cells in its epithelium to protect the intestinal barrier and help maintain intestinal equilibrium. The portal vein system transports nutrients and toxins from the intestine to the liver for processing. Concurrently, the liver excretes bile acids into the intestine via the biliary system, metabolizing them in collaboration with microorganisms. This establishes a reciprocal regulatory relationship between the liver and the intestine that manages metabolic byproducts, nutrients, and bile acids— referred to as the gut‐liver axis [13, 14]. The gut microbiota plays vital roles in host defense against pathogens, immune modulation, metabolic pathways, and gut‐liver circulation. Imbalances in this microbiota can lead to choline deficiency, increased intestinal permeability, reduced SCFA synthesis, changes in bile acid composition, resulting in liver inflammation, fibrosis, and metabolic disruptions. Ongoing research focuses on the relationship between liver metabolism and the gut microbiota, emphasizing biochemical reactions and small molecules. Yet, the influence of CD8^+^ T cell deficiency on gut microbiota diversity, liver biochemistry, and their connections remains unclear. This study explores changes in gut microbiota and liver biochemistry due to CD8^+^ T cell deficiencies, elucidating the link between the immune system (CD8^+^ T cells) and the gut‐liver axis.

## 2 Materials and Methods

### 2.1 Experimental Animals, Grouping and Sample Collection Protocols

Female C57BL/6J mice aged 6–8 weeks and C57BL/6J‐Cd8aem1cyagen mice (hereinafter referred to as CD8^+^ T cell‐deficient mice) were purchased from Cyagen Technologies Co. Ltd. All mice used in this experiment were of the C57BL/6J strain. All mice were housed in an SPF‐grade animal facility under the following conditions: temperature 22°C ± 2°C, relative humidity 40%–60%, 12 h/12 h light/dark cycle (lights on at 8:00 a.m.), 15 air changes per hour, and HEPA‐filtered air supply. Mice were kept in individually ventilated cages (IVCs) with autoclaved corncob bedding. Each cage housed 6 mice. Standard rodent chow and autoclaved tap water were provided ad libitum. Environmental enrichment included sterile nesting material (cotton squares) and a red polycarbonate tunnel for shelter. Cages were changed once per week inside a biosafety cabinet. All procedures involving opening the cages were performed under a laminar flow hood. After a 1‐week acclimatization period, the experiments began. All experiments were approved by the Ethics Committee of Ningxia Medical University. The mice were divided into three groups: the normal control group, the CD8^+^ T cell‐deficient group, and the CD8^+^ T cell reinfusion group, named C, CH, and ZG, respectively, with six mice in each group. The CD8^+^ T cell reinfusion group involved isolating CD8^+^ T cells from each normal group mouse and injecting them into each CD8^+^ T cell‐deficient mouse via the tail vein. Each normal group mouse corresponded to one CD8^+^ T cell‐deficient mouse, with a total of four reinfusions, each spaced 3 days apart. Four weeks after the final transfusion, feces were collected from mice in each group, and liver tissue from each group was isolated and frozen in liquid nitrogen for future use.

### 2.2 DNA Extraction From Mouse Tail Tissue

At 2 weeks of age, cut approximately 0.2 cm of the mouse's tail tip and place it in an EP tube free of DNA contamination. Use surgical scissors to chop it into small pieces, then add 200 μL of buffer solution GA and shake until thoroughly suspended. Add 20 μL of Proteinase K, mix thoroughly, and place in a 56°C water bath for approximately 5–6 h until the tissue dissolves. Add 200 μL of buffer GB, mix thoroughly, and place in a 70°C water bath for 10 min until the solution becomes clear. Add 200 μL of anhydrous ethanol, vortex for 15 s until thoroughly mixed, and briefly centrifuge to remove water droplets from the inner wall of the tube cap. Add the solution obtained from the above steps to an adsorption column and place it in a collection tube. Centrifuge at 12,000 rpm for 30 s at 4°C, discard the waste liquid in the tube, and place the adsorption column back into the collection tube. Add 500 μL of buffer GD to the adsorption column, centrifuge at 12,000 rpm for 30 s at 4°C, discard the waste liquid in the tube, and place the adsorption column back into the collection tube. Add 600 μL of wash solution PW to the adsorption column, at 12,000 rpm for 30 s at 4°C, discard the waste liquid in the tube, and place the adsorption column back into the collection tube. Repeat twice. Place the adsorption column back into the collection tube, centrifuge at 12,000 rpm for 2 min at 4°C, discard the waste liquid in the collection tube, and leave the adsorption column at room temperature for 5 min to dry any residual PW from the material. Transfer the adsorption column to a new 1.5 mL EP tube. Add 20 μL of elution buffer to the middle of the adsorption column membrane, let it sit at room temperature for 5 min, centrifuge at 12,000 rpm for 2 min, remove the liquid from the EP tube, and add it again to the middle of the adsorption column. Centrifuge again to completely elute the DNA from the membrane. The liquid in the centrifuge tube is the extracted genomic DNA.

### 2.3 PCR Amplification Reaction

All primers were provided and validated by Cyagen Biosciences, and all primers were synthesized by Sangon Biotech (Shanghai) Co. Ltd. The primer sequences are listed in Table 1 below. PCR amplification reaction system: 12.5 μL 2 × Taq PCR Master Mix, 1 μL primer, 1.5 μL DNA template, and ddH_2_O added to a total volume of 25 μL. PCR amplification program: 94°C pre‐denaturation for 3 min, 94°C denaturation for 30 s, 60°C annealing for 35 s, 72°C extension for 35 s, 35 cycles, and 72°C for 5 min to terminate the reaction.

**Table 1:** Primer sequences for gene identification in CD8^+^ T cell‐deficient mice.

Primers		Sequence (5′−3′)
CD8F1		GTCCCTTCCAAGTGTTTTCTCTTC
CD8F2		CCAGACCTTCAGAGAAAATTGTGTA
CD8R1		GCAGTTCTCCTGACTTTGATTTGTT

### 2.4 Isolation of Mouse CD8^+^ T Cells

Aseptically isolate the spleen, place it on a 70 μm filter membrane and gently grind it while adding sample dilution to make 5 mL of cell suspension, blowing and mixing; add 5 mL of mouse splenic lymphocyte isolate in a 15 mL centrifuge tube, then tilt the tube and slowly add the cell suspension from the previous step in two batches, 450*g*, 22°C, centrifugation for 20 min; after centrifugation was completed it was divided into four layers, the upper liquid was discarded, and the white membrane layer was washed twice. After centrifugation, the cells were divided into 4 layers, and the upper layer of liquid was discarded. The white membrane layer was carefully aspirated into another clean centrifuge tube, and 10 mL of washing solution was added, 350*g*, 22°C, centrifuged for 10 min, and washed twice; finally, the cells were resuspended with complete medium for counting. The separated cells were incubated for 5 min at 4°C with 40 μL of magnetic bead sorting buffer and 10 μL of Biotin‐Antibody Cocktail for every 1 × 10^7^ of cells, and then 30 μL of magnetic bead sorting buffer and 20 μL of Anti‐Biotin MicroBeads (IgG1) were added, and the cells were incubated for 10 min at 4°C with the magnetic bead sorting buffer and 20 μL of Anti‐Biotin MicroBeads (IgG1). mins; the magnetic bead sorting column was fixed on a magnetic stand with a beaker filled with ice underneath, the incubated cell suspension was diluted, and 2–3 mL of magnetic bead sorting buffer was added to the sorting column to fully wet the column, and then the magnetic bead sorting was carried out by adding the buffer and cell suspension at the same time; the cells were centrifuged for 10 min at 350*g* at 4°C, and after centrifugation was completed, the cells were completely resuspended in 1640 complete After centrifugation, the cells were resuspended in 1640 complete medium.

### 2.5 Agarose Gel Electrophoresis

Add 1 g of agarose to 100 mL of TAE buffer, mix thoroughly, then heat in a microwave oven until boiling three times until it becomes clear and transparent. Allow it to cool slightly, add 4 μL of nucleic acid dye, gently shake to mix evenly, and pour the solution into the gel casting mold with the electrophoresis comb inserted. After 30 min of gel solidification, remove the comb, carefully remove the gel from the gel casting apparatus, and place it in the electrophoresis chamber. Add 4 μL of the PCR amplification product obtained above to each well, and add 4 μL of 2000 bp DNA marker to the end wells. Set the voltage to 120 V and the electrophoresis time to 40 min. After electrophoresis, place the gel under a UV imaging system for photography and observation, record the results, and analyze the mouse genotype.

### 2.6 Flow Cytometry

The CD8^+^ T cell suspension obtained from the magnetic bead sorting in the above steps, 500*g*, 4°C, centrifugation for 5 min, discard the supernatant, add Buffer Ⅱ 3 mL for full resuspension and mixing, 350*g*, 4°C, centrifugation for 8 min, discard the supernatant, add the antibody, resuspension and mixing, incubated for 30 min, protected from light, add 3 mL Buffer Ⅱ, mixing, 350*g*, 4°C, centrifugation for 8 min, discard the supernatant, Buffer Ⅱ and paraformaldehyde fixative made into a mixture at a ratio of 500:1, take 1 mL to add and fully mix, and wait for the upper machine. 4°C, centrifuged for 8 min, discard the supernatant, Buffer Ⅱ and paraformaldehyde fixative made at the ratio of 500:1 mixture, take 1 mL to add and mix well, waiting for the machine. At the same time, make a single dye tube and an empty dye tube for compensation adjustment, and analyze the data using FlowJo 10.8.1.

### 2.7 Sample Preparation and Sequencing Analysis

Mouse feces were collected and placed in freezing tubes and stored at −80°C for subsequent use. Sample DNA was first extracted (TIANGEN, DP328, China), following the steps of TIANGEN Fecal Genomic DNA Extraction Kit. After DNA extraction, the concentration of nucleic acids was measured by spectrophotometry, followed by amplification and electrophoresis of the amplified PCR products using agarose at 1.8% concentration, and sequencing. In the second step, PCR was performed using the PCR product from the first step as a template, and sequencing was performed using the Illumina Novaseq. 6000 platform.

Isolated mouse livers were placed in freezing tubes and stored at −80°C for subsequent use. 50 mg of sample was added to 1000 μL of acetonitrile: methanol: water (2:2:1). After gently vortexing for 30 s, the sample was homogenized at 45 Hz for 10 min, and then ultrasonicated in an ice‐water bath for 5 min. The samples were then placed at −20°C for 1 h. The samples were removed and centrifuged at 4°C for 15 min at 12,000 rpm/min. 500 μL of the supernatant was removed in an EP tube and the extract was dried in a vacuum concentrator. The remaining sample was vortexed with 160 μL of L acetonitrile: water (1:1, v/v) for 30 s. The samples were sonicated in an ice‐water bath for 10 min, and centrifuged again at 4°C, 12000 rpm/min for 15 min, and the supernatant was taken out and put into new Ep tubes, and finally, 10 μL of each sample was taken out and assayed on the machine.

### 2.8 Diversity Analysis

CCS sequences were derived from the raw data, CCS sequences were identified, length filtered, chimeras were removed and EffectiveCCS was obtained, EffectiveCCS sequences were clustered/denoised and OTUs were classified. The Alpha diversity index of the samples was assessed using QIIME2 software. Differences in Alpha diversity indices between groups were assessed using *t*‐test. Beta diversity analysis was carried out using QIIME software to compare the degree of similarity existing in species diversity among different samples. Beta diversity analysis was performed based on the Bray–Curtis algorithm.

### 2.9 LC‐MS/MS Analysis

A Waters Xevo G2‐XS QTOF high‐resolution mass spectrometer was used to collect and analyze the data. The mass spectra were characterized by a low collision energy of 2 V, a high collision energy range of 10–40 V, and a scanning frequency of 0.2 s. The ion source parameters were as follows: capillary voltage: 2000 V (positive ion mode) or −1500 V (negative ion mode); cone voltage: 30 V; ion source temperature: 150°C; desolvation gas temperature: 500°C; backwash gas flow rate: 50 L/h; and desolvation gas flow rate: 800 L/h. Raw data were collected using MassLynx v4.2, and peaks were extracted using the Progensis QI software was used for data processing operations such as peak extraction and peak comparison. The online Metlin database and the self‐constructed BioMark were characterized using Progensis QI software.

### 2.10 Statistical Analysis

All data were analyzed using GraphPad Prism 8.0 (GraphPad Software) and expressed as mean ± standard deviation. The independent two‐sample *t*‐test was used for comparisons between two groups. One‐way or two‐way analysis of variance (ANOVA) followed by post‐hoc multiple comparison tests was applied to datasets with more than two groups to evaluate statistical differences between any two groups. A *p* value less than 0.05 was considered statistically significant.

### 2.11 Statement

Our research does not involve any content created or generated by artificial intelligence and is entirely original. We have adhered to the relevant guidelines provided on the journal's website.

## 3 Results and Analysis

### 3.1 Construction of Animal Models

As shown in Figure 1a,b,e, the CD8^+^ T cell‐deficient mice used in this study were completely homozygous defective strains. The proportion of CD8^+^ T cells in normal C57BL/6 J mice was 41.9%, while in defective mice it was only 0.99%. The results of Figure 1c,d show that on the first day after the quantitative infusion of CD8^+^ T cells into CD8^+^ T cell‐deficient mice, the proportion recovered to 16.4%. By the third day, it decreased significantly to 0.86%, and the difference was statistically significant. Therefore, based on the monitoring data from flow cytometry, this study adopted a strategy of repeatedly reinfusing CD8^+^ T cells every 3 days to ensure that the level of CD8^+^ T cells in mice remained stable during the experiment. Figure 1f shows the results of mouse genotype identification. Lanes 1 and 6 are DNA markers. In lanes 2 and 3, a 578 bp band was amplified only in lane 3; these mice were identified as wild‐type individuals from the CD8^+^ T cell‐deficient strain. In lanes 4 and 5, a 436 bp band was amplified only in lane 4; these mice were identified as homozygous CD8^+^ T cell‐deficient mice.

**Figure 1:**
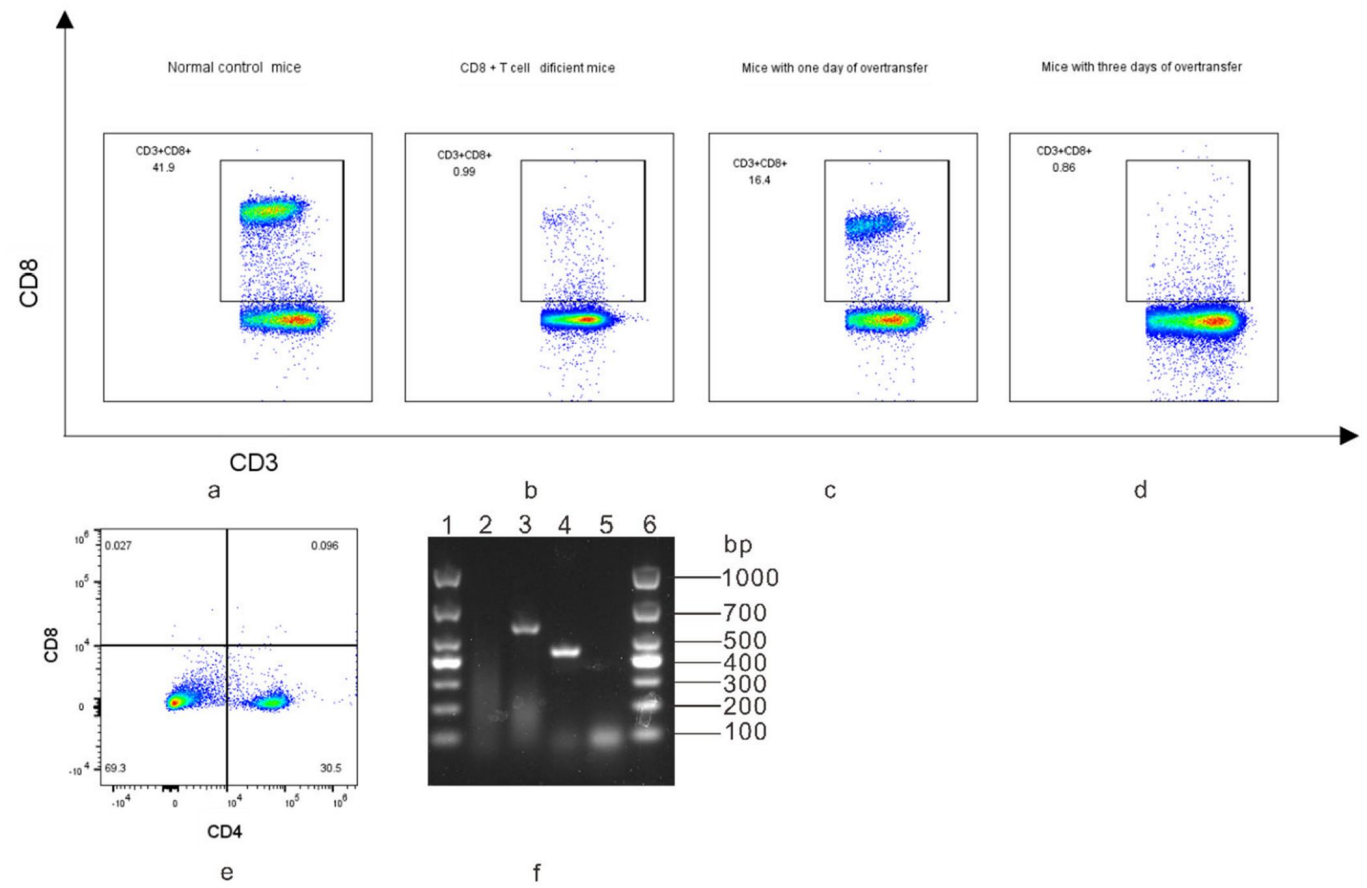
The effect of adoption transfer on the proportion of CD8^+^ T cells and the results of genotype identification in CD8^+^ T cell‐deficient mice. *Note:* (a) CD3⁺CD8⁺ flow cytometry plot of normal control mice; (b) CD3⁺CD8⁺ flow cytometry plot of CD8⁺ T cell‐deficient mice; (c) CD3⁺CD8⁺ flow cytometry plot on day 1 after adoptive transfer of CD8⁺ T cells; (d) CD3⁺CD8⁺ flow cytometry plot on day 3 after adoptive transfer of CD8⁺ T cells; (e) representative CD4/CD8 flow cytometry plot confirming the absence of CD8⁺ T cells in deficient mice (f) PCR genotype identification, with lanes 1 and 6 as DNA markers, lane 3 showing a 578‐bp band, and lane 4 showing a 436‐bp band.

### 3.2 Analysis of Gut Microbial Diversity

Venn analysis at the OTU level was performed on fecal samples from the normal control, CD8^+^ T‐cell‐deficient and CD8^+^ T‐cell‐return groups, as shown in Figure 2a, resulting in 5337 OTUs (Operational Taxonomic Units), with 2263 OTUs in the control group, 2310 OTUs in the CD8^+^ T‐cell‐deficient group and CD8^+^ T‐cell back group had OTUs 2096. Figure 2b displays the dilution curve, while Figure 2c illustrates the hierarchical abundance curve, suggesting the reliability of the sequencing results for subsequent analysis of community structure disparities among samples.

**Figure 2:**
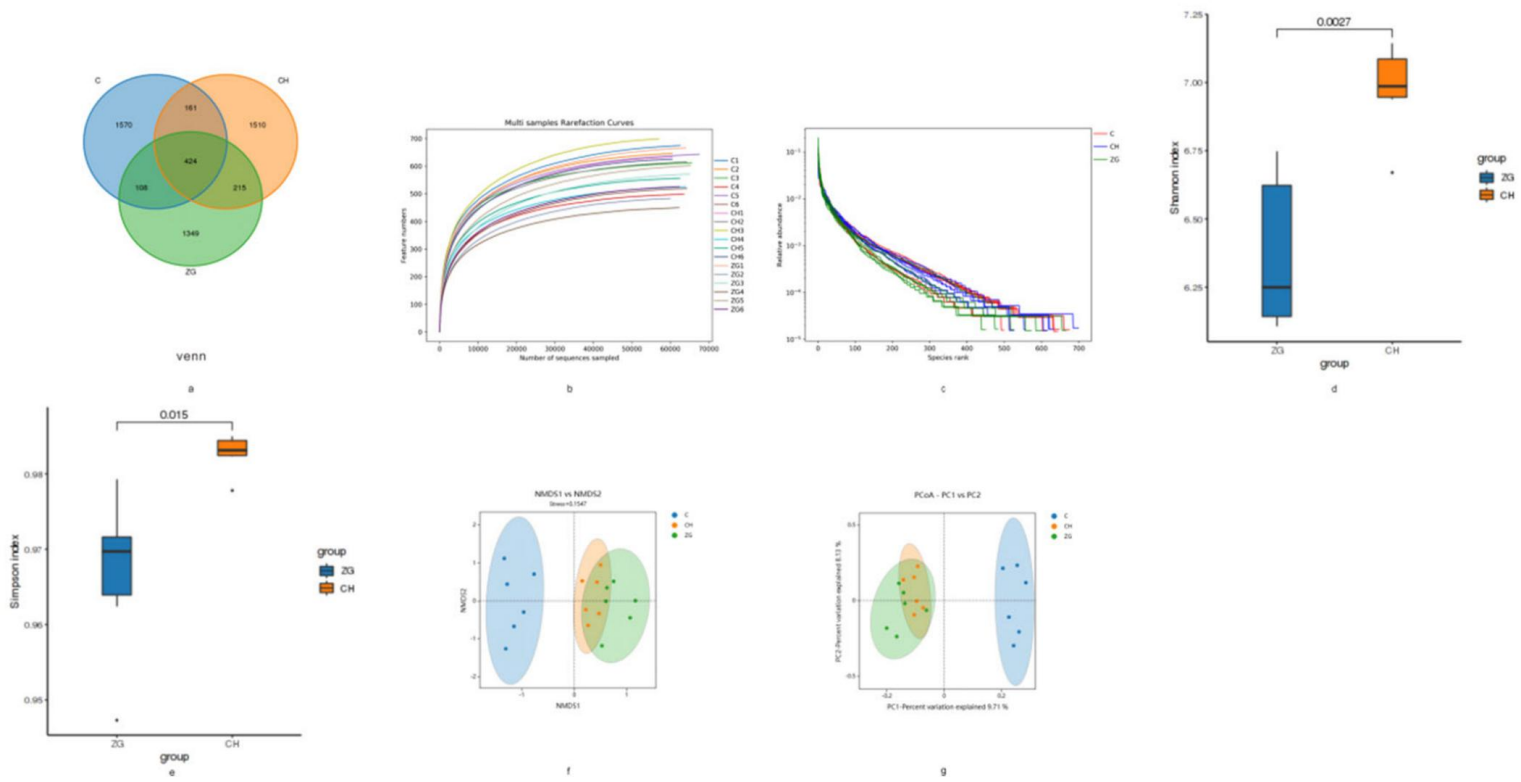
Gut microbial diversity. *Note:* (a) Venn analysis of the gut microbiota of control, CD8^+^ T‐cell‐deficient, and CD8^+^ T‐cell‐returned mice; (b) Dilution curves for each sample in control, CD8^+^ T‐cell‐deficient and CD8^+^ T‐cell‐returned groups; (c) Rank‐Abundance plots for each sample in control, CD8^+^ T cell‐deficient and CD8^+^ T cell‐returned groups; (d) and (e) represent box plots of Shannon and Simpson indices for the CD8^+^ T‐cell‐deficient group as well as the CD8^+^ T‐cell‐return group, respectively; (f) NMDS analysis plots of the control group, the CD8^+^ T‐cell‐deficient group, and the CD8+ T‐cell‐return group;(g) PCoA principal coordinates analysis of the control, CD8^+^ T‐cell‐deficient, and CD8^+^ T‐cell‐return groups.

The species diversity within a single sample was studied by Alpha diversity analysis. Where Ace index measures the species abundance that is, the number of species, Shannon and Simpson indices are used to measure the species diversity. In this study, the Ace index and Shannon and Simpson indices were used to evaluate the abundance and diversity of mouse gut microbiota, and Good's Coverage value was used to indicate the sequencing depth of the samples. As shown in Table 2, the Good's Coverage values of each group were all 1, indicating that the sequencing depth met the requirements of subsequent experiments. According to Table 2 and shown in Figure 2d,e, compared with the CD8^+^ T‐cell‐deficient group, the Simpson index and Shannon index of the CD8^+^ T‐cell‐returned group were reduced, and the difference was statistically significant (*p* < 0.05), indicating that the species diversity of the gut microbiota of the mice was decreased after CD8^+^ T‐cell‐returned. As shown in Figure 2f,g below, the results of PCoA principal coordinate analysis and NMDS analysis suggested that there was a significant difference in the microbial community structure of the samples from the normal control group and the CD8^+^ T‐cell‐deficient group.

**Table 2:** Alpha diversity comparison (*n* = 6, *x̄*±s).

Group	ACE	Simpson	Shannon	Good's Coverage	
Normal control group	603.55 ± 74.50	0.98 ± 0.01	6.85 ± 0.40	1	
CD8^+^ T‐cell‐deficient group	613.24 ± 62.11	0.98 ± 0.00^a^	6.97 ± 0.17^b^	1	
CD8^+^ T cell return group	555.04 ± 80.41	0.96 ± 0.01^a^	6.37 ± 0.30^b^	1	

*Note:* Data are expressed as mean ± standard error. ‘a’ represents the Simpson index of the CD8^+^ T cell deficiency group and the CD8^+^ T cell reinfusion group. ‘b’ represents the Shannon index of the CD8^+^ T cell deficiency group and the CD8^+^ T cell reinfusion group. *p* < 0.05.

### 3.3 Abundance of Gut Microbiota

To analyze the causes of differences in the relative abundance of microbial flora and to investigate the impact of CD8^+^ T cell deficiency on the gut microbiota, we quantified the relative abundance of gut microbiota at different taxonomic levels in order to identify characteristic microorganisms at each level. As shown in Figure 3a,d, the top 10 most abundant bacteria at the phylum level are presented. Among these, *Bacteroidetes* and *Firmicutes* were the dominant phyla, exhibiting comparable relative abundances. As shown in Figure 3b,e, the top 10 most abundant bacteria at the family level are displayed. *Muribaculaceae* and *Lachnospiraceae* were the dominant families, with *Muribaculaceae* showing the highest relative abundance, followed by *Lachnospiraceae*. As shown in Figure 3c,f, the top ten most abundant bacteria at the genus level are shown. The *Lachnospiraceae_NK4A136_group* and *unclassified Muribaculaceae* were the dominant groups, with unclassified *Muribaculaceae* exhibiting the highest relative abundance, followed by the *Lachnospiraceae_NK4A136_group*.

**Figure 3:**
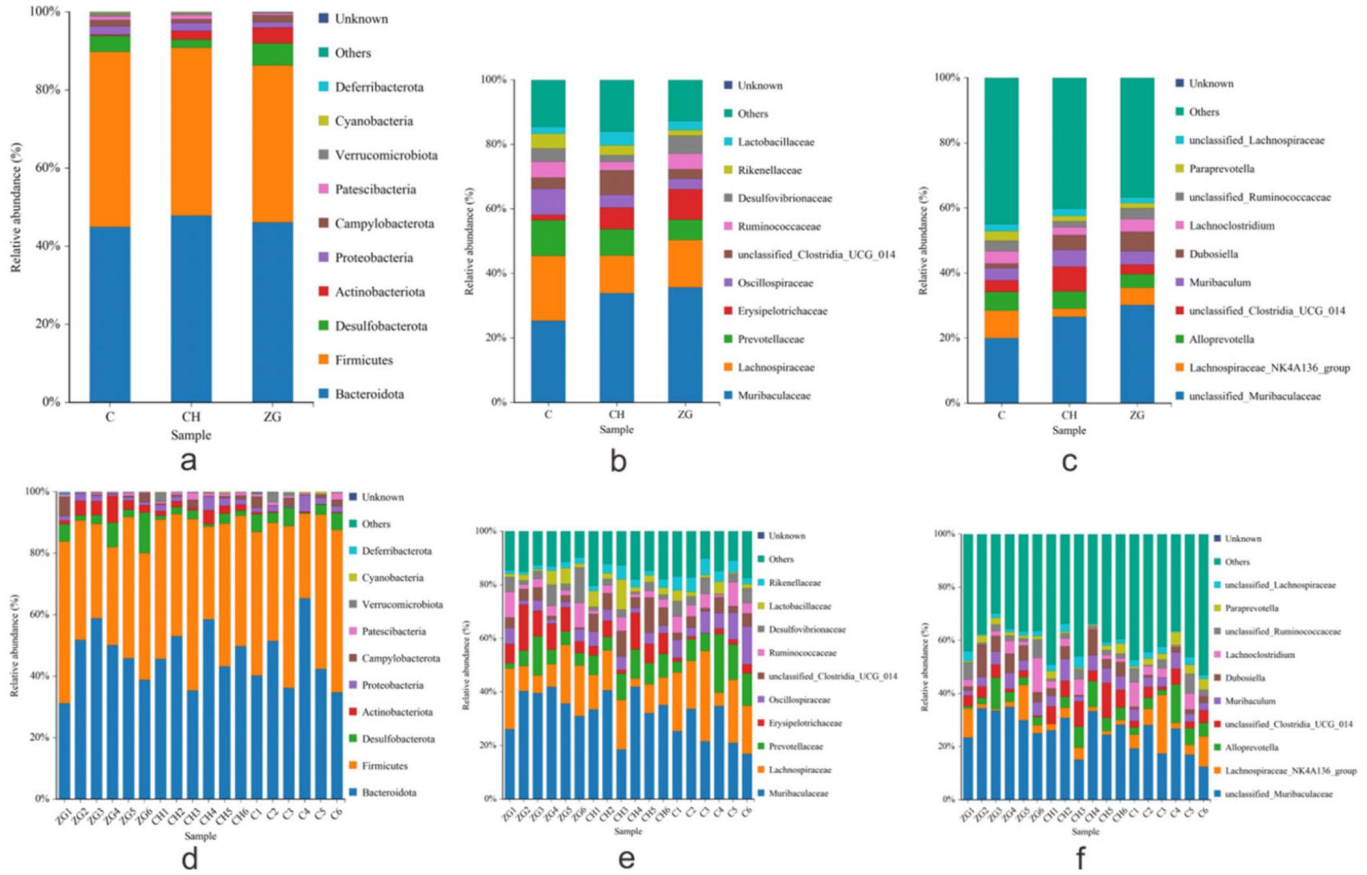
Histogram of species abundance (*n* = 6). *Note:* (a), (b), and (c) represent the relative abundance of species among different groups at the phylum, family, and genus levels, respectively. (d), (e), and (f) are the relative abundance of species among different samples at the phylum, family, and genus levels, respectively.

### 3.4 LEfSe Analysis

The analysis by LEfSe allows for the discovery of complex biological markers and the exploration of genomic traits associated with genes and metabolism to differentiate between diverse biological states. Linear discriminant analysis (LDA) was applied to evaluate the influence of each component's levels on the observed variations. Figure 4a and present the distribution of gut microbiota species in different mouse groups, as examined through LEfSe. By applying an LDA threshold of ≥ 3.5, a total of 24 unique bacterial species were recognized. Variations in bacterial species among different groups are mainly found within the *Firmicutes*, *Bacteroidetes*, *Proteobacteria*, and *Actinobacteriota* phyla. The results of the LEfSe analysis further confirm the observations on the proportions of microbial flora.

**Figure 4:**
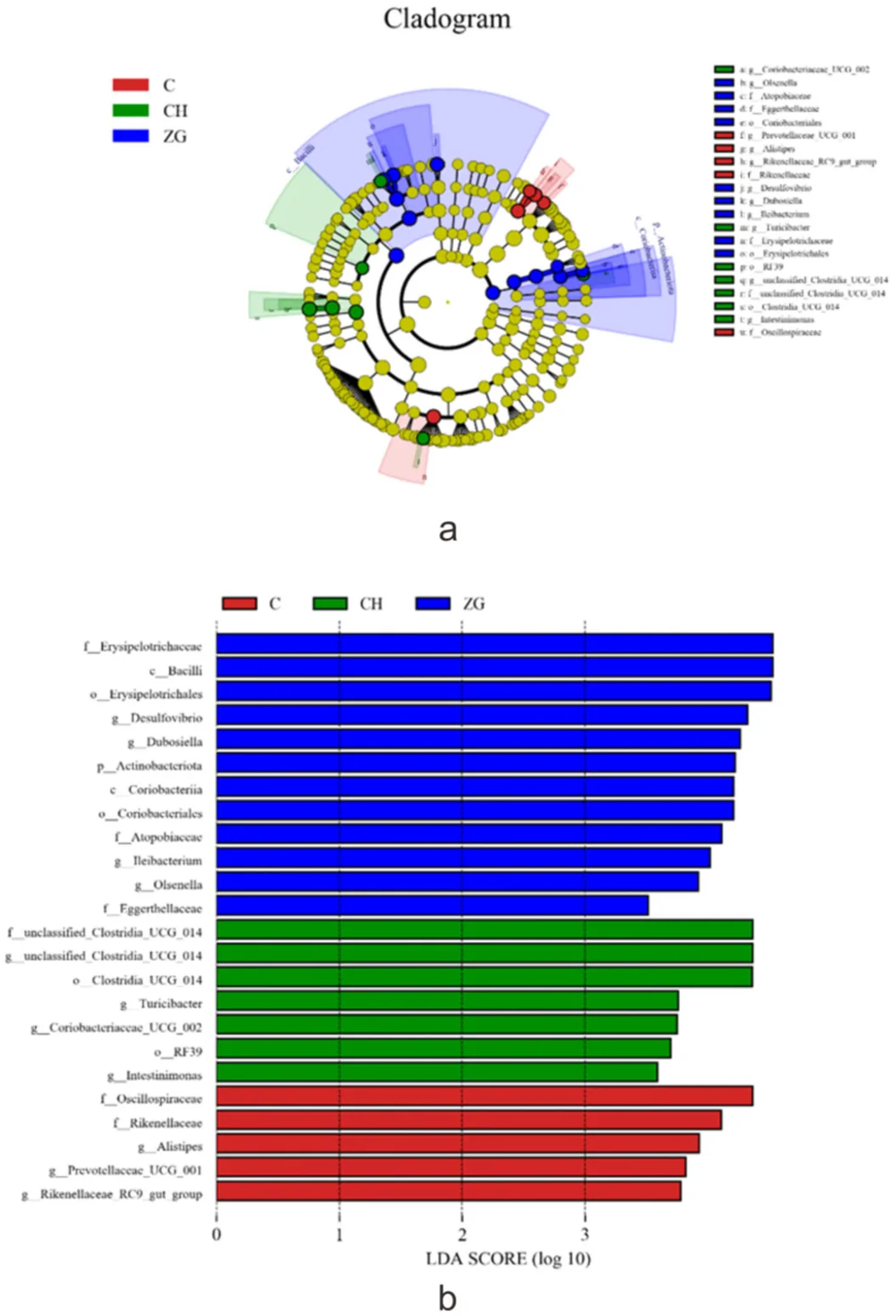
Differential analysis of LEfSe in the gut microbiota of mice from different groups. *Note:* (a) Branching diagram of species evolution of mouse gut microbiota in different groups; (b) Histogram of distribution of LDA values of marker species of mouse gut microbiota in different groups.

### 3.5 Analysis of Differentially Expressed Gut Microbiota

To further investigate the differences in the gut microbiota between treated mice and control mice, Metastats software was used to perform pairwise comparisons of species abundance between groups with *t*‐test analysis. Species contributing to compositional differences between the two groups were screened according to *p* values. Intergroup significance analysis was performed at the family level. The results of the study showed that at the family level, compared with normal control mice, CD8^+^ T cell‐deficient mice *unclassified_Clostridia_UCG_014*, *unclassified_RF39*, *uncultured_Lachnospiraceae_bacterium*, *uncultured_Erysipelotrichaceae_bacterium*, *bacterium_endosymbiont_of_Onthophagus_Taurus* showed an upward trend (*p* < 0.05); compared with mice in the CD8^+^ T‐cell‐deficient group, mice in the CD8^+^ T‐cell‐return group *unclassified_Clostridia_UCG_014*, *unclassified_RF39*, *uncultured_Lachnospiraceae_bacterium*, *uncultured_Erysipelotrichaceae_bacterium bacterium_endosymbiont_of_Onthophagus_Taurus* showed a decreasing trend *(p* < 0.05). At the genus level, compared with normal control mice, CD8^+^ T cell‐deficient mice *unclassified_Clostridia_UCG_014*, *Intestinimonas*, *unclassified_RF39*, *uncultured_Lachnospiraceae_bacterium*, *uncultured_Muribaculaceae_bacterium*, *Family_XIII_AD3011_group*, *uncultured_Erysipelotrichaceae_bacterium*, *Catabacter*, *Vicinamibacter*, *Mesorhizobium*, *unclassified_bacterium_endosymbiont_of_Onthophagus_Taurus*, *Catellatospora*, *Stomatobaculum* showed an increasing trend (*p* < 0.05); compared to the CD8^+^ T cell‐deficient group, the CD8^+^ T‐cell‐return group mice *unclassified_Clostridia_UCG_014*, *Intestinimonas*, *unclassified_RF39*, *uncultured_Lachnospiraceae_bacterium*, *uncultured_ Muribaculaceae_bacterium*, *Family_XIII_AD3011_group*, u*ncultured_Erysipelotrichaceae_bacterium*, *Catabacter*, *Vicinamibacter*, *Mesorhizobium*, *unclassified_bacterium_endosymbiont_of_Onthophagus_Taurus*, *Catellatospora*, *Stomatobaculum* showed a decreasing trend (*p* < 0.05) as shown in Tables 3, 4, 5, 6. As can be seen from the table, the differential microbiota derived in this study is approximately the same as the results of LEfSe analysis.

**Table 3:** Differences in family levels between normal control mice and CD8^+^ T cell‐deficient mice Gut microbiota.

Differential microbiota	Mean(C)	Mean(CH)	*p* value	Variation trend
*bacterium_endosymbiont_of_Onthophagus_Taurus*	0	0.000013	0.02821277	↑
*uncultured_Erysipelotrichaceae_bacterium*	0	0.0000138	0.02821277	↑
*uncultured_Lachnospiraceae_bacterium*	0.00048431	0.00219689	0.01777451	↑
*unclassified_RF39*	0.00191912	0.00371293	0.00182353	↑
*unclassified_Clostridia_UCG_014*	0.03518203	0.07644031	0.00840196	↑

**Table 4:** Gut microbiota of CD8^+^ T‐cell‐deficient group mice after backfilling CD8^+^ T cells and CD8^+^ T‐cell‐deficient group mice with differences in the level of family.

Differential microbiota	Mean(ZG)	Mean(CH)	*p* value	Variation trend
*unclassified_Clostridia_UCG_014*	0.029011941	0.0764403	0.00333684	↓
*unclassified_RF39*	0.00136219	0.0037129	0.00057895	↓
*uncultured_Lachnospiraceae_bacterium*	0.000137732	0.0021969	0.00522105	↓
*uncultured_Erysipelotrichaceae_bacterium*	0	0.0000138	0.02921718	↓
*bacterium_endosymbiont_of_Onthophagus_Taurus*	0	0.000013	0.02921718	↓

**Table 5:** Genus level differences between normal control mice and CD8^+^ T‐cell deficient mice Gut microbiota.

Differential microbiota	Mean(C)	Mean(CH)	*p* value	Variation trend
*Stomatobaculum*	0	0.0000129	0.02821183	↑
*Catellatospora*	0	0.000013	0.02821183	↑
*unclassified_bacterium_endosymbiont_of_Onthophagus_Taurus*	0	0.000013	0.02821183	↑
*Mesorhizobium*	0	0.000013	0.02821183	↑
*Vicinamibacter*	0	0.0000133	0.02821183	↑
*Catabacter*	0	0.0000135	0.02821183	↑
*uncultured_Erysipelotrichaceae_bacterium*	0	0.0000138	0.02821183	↑
*Family_XIII_AD3011_group*	0.0001514	0.000479969	0.000605	↑
*uncultured_Muribaculaceae_bacterium*	0.000978	0.001711462	0.01277	↑
*uncultured_Lachnospiraceae_bacterium*	0.0004843	0.002196894	0.016915	↑
*unclassified_RF39*	0.0019191	0.003712934	0.00195	↑
*Intestinimonas*	0.0055221	0.013518823	0.02915	↑
*unclassified_Clostridia_UCG_014*	0.0351817	0.076440307	0.007775	↑

**Table 6:** Gut microbiota of CD8^+^ T‐cell‐deficient group mice after backfilling with CD8^+^ T‐cells differed from CD8^+^ T‐cell‐deficient group mice at genus level.

Differential microbiota	Mean(ZG)	Mean(CH)	*p* value	variation trend
*unclassified_Clostridia_UCG_014*	0.02901181	0.07644031	0.00374033	↓
*Intestinimonas*	0.00484103	0.01351882	0.0220884	↓
*unclassified_RF39*	0.00136217	0.00371293	0.00061326	↓
*uncultured_Lachnospiraceae_bacterium*	0.00013773	0.00219689	0.0048674	↓
*uncultured_Muribaculaceae_bacterium*	0.00067683	0.00171146	0.00122099	↓
*Family_XIII_AD3011_group*	0.00012574	0.00047997	0	↓
*uncultured_Erysipelotrichaceae_bacterium*	0	0.0000138	0.02921679	↓
*Catabacter*	0	0.0000135	0.02921679	↓
*Vicinamibacter*	0	0.0000133	0.02921679	↓
*Mesorhizobium*	0	0.000013	0.02921679	↓
*Catellatospora*	0	0.000013	0.02921679	↓
*unclassified_bacterium_endosymbiont_of_Onthophagus_Taurus*	0	0.000013	0.02921679	↓
*Stomatobaculum*	0	0.0000129	0.02921679	↓

### 3.6 Screening for Differential Metabolites in the Liver

To explore the changes of differentially expressed metabolites in the livers of mice in the CD8^+^ T‐cell‐deficient group and normal control group as well as mice in the CD8^+^ T‐cell‐return group, we screened for differentially expressed metabolic molecules based on the conditions of VIP > 1 and *p* < 0.05 as well as |log2FC|> 2. As shown in Figure 5a, a total of 121 metabolic molecules were screened in the CD8^+^ T‐cell‐deficient group of mice compared with the control mice, of which 64 molecules were up‐regulated and 57 molecules were down‐regulated in the CD8^+^ T‐cell‐deficient group of mice compared with the control mice, and the top 15 metabolites with the greatest metabolic differences are shown in Tables 7 and 8. The metabolic levels of 123 sub‐metabolic molecules were changed after infusing CD8^+^ T cells back into the mice compared with the control mice as shown in Figure 5b, of which 48 metabolic molecules were up‐regulated and 75 were down‐regulated in comparison with normal mice, and the top 15 metabolites with the greatest metabolic differences are shown in Tables 9 and 10.

**Figure 5:**
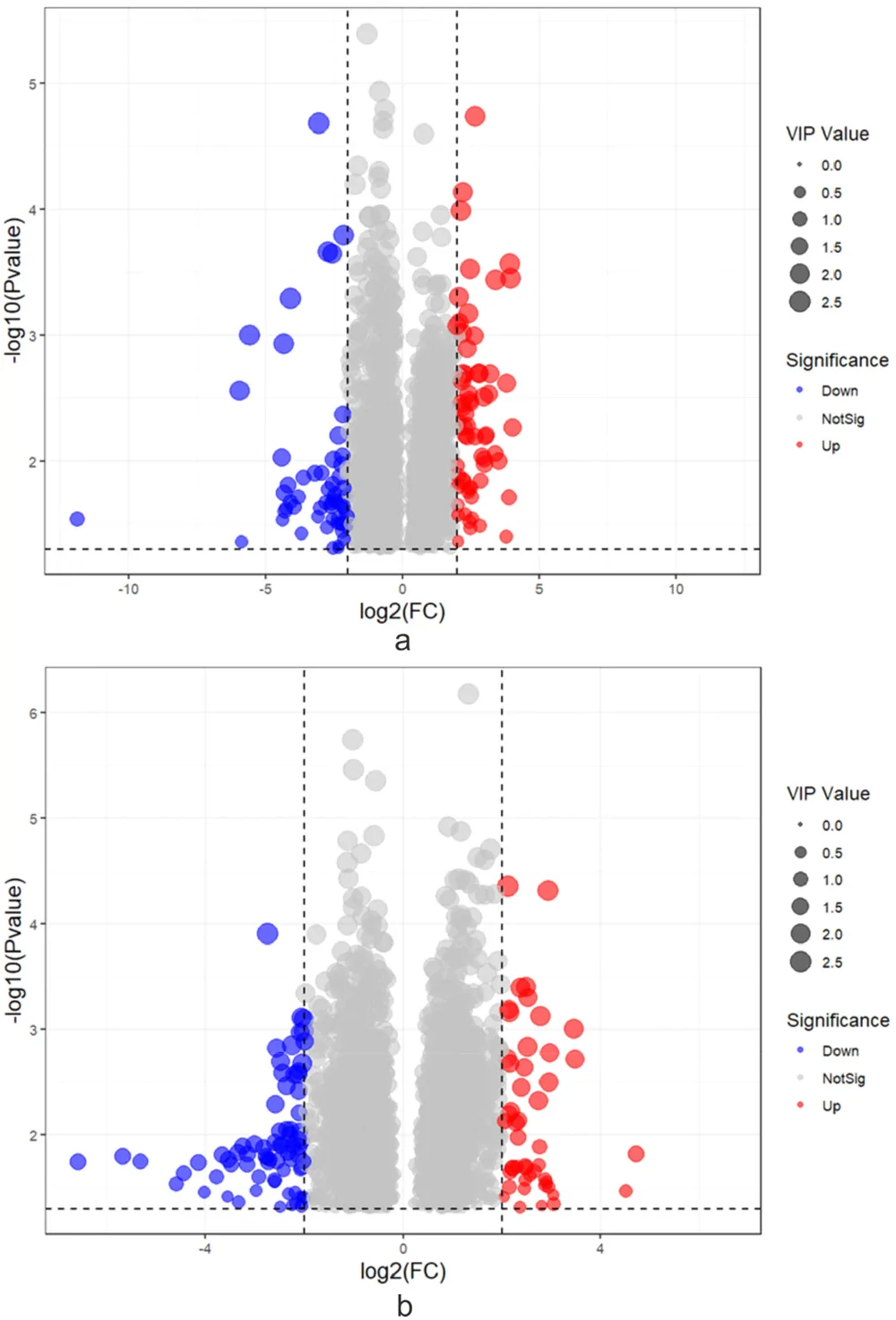
Volcano map of differential metabolites. (a) CD8^+^ T‐cell‐deficient group vs. control group; (b) CD8^+^ T‐cell‐return group vs. control group.

**Table 7:** Metabolic molecules up‐regulated at expression levels in CD8^+^ T cell‐deficient mice compared to normal mice.

name	Mean(C)	Mean(CH)	log2FC	*p* value	VIP
3′‐Hydroxy‐HT2 toxin	9.750755	159.2754	4.029866	0.005421	1.716079
1‐(6Z,9Z,12Z‐octadecatrienoyl)‐glycero‐3‐phosphate	29.08712	457.5682	3.975535	0.000353	1.942829
metenolone acetate	13.18281	202.045	3.937947	0.000273	1.957312
7‐Deaza‐2′‐deoxyguanosine	8.499313	127.1658	3.90322	0.019523	1.537454
4‐Hydroxynonenal	76.25141	1087.83	3.834546	0.002428	1.817364
Ocfentanil	64.81068	897.1589	3.79106	0.039982	1.386175
Zeinoxanthin	198.7626	2320.5	3.545318	0.010052	1.631973
PE(15:0/P‐16:0)	38.84002	413.5018	3.412278	0.000366	1.935832
Avermectin B1a	36.33318	378.4869	3.380884	0.008648	1.616058
Bialaphos	23.35624	215.8881	3.208404	0.002052	1.801943
Dapdiamide C	238.9035	2126.346	3.153876	0.002932	1.759244
C20915	19.67389	163.762	3.057247	0.006225	1.632494
3,7‐Dimethyl‐5‐octene‐1,7‐diol 1‐glucoside	12.56154	103.1872	3.038179	0.006457	1.670814
6‐[(2S,3S,4R,5R)−5‐(6‐Aminopurin‐9‐yl)−3,4‐dihydroxyoxolan‐2‐yl]oxane‐2,5‐dione	16.59519	134.3275	3.016918	0.0107	1.63202
Naftidrofuryl	34.33975	271.723	2.984185	0.009597	1.62033

**Table 8:** Down‐regulation of metabolic molecules at expression levels in CD8^+^ T cell‐deficient mice compared to normal mice.

name	Mean(C)	Mean(CH)	log2FC	*p* value	VIP
Pyrimidodiazepine	44880	11.96647	11.8729	0.029085	1.473098
N‐Acetoxymethylflindersine	1739.2	28.46433	5.93312	0.002776	1.850249
Dihydro‐beta‐erythroidine	11308.92	193.0657	5.87222	0.044151	1.348863
trans‐2‐Dodecenoylcarnitine	205.6762	4.28992	5.58328	0.001005	1.934206
Endralazine	585.7518	27.67011	4.40389	0.009403	1.708294
Virol A	248.6604	11.82518	4.39424	0.029591	1.391264
Rokitamycin	134514.8	6652.053	4.33782	0.017854	1.605511
[(2S,3R)−2‐Amino‐3‐hydroxybutanoyl] (2S)‐pyrrolidine‐2‐carboxylate	2493.326	124.9172	4.31903	0.001173	1.92515
Crispolide	3134.36	158.832	4.3026	0.025003	1.49193
14,15‐DHET	476.6829	25.44983	4.2273	0.02387	1.44523
1‐Cinnamoylpiperidine	3994.506	219.0709	4.18855	0.015554	1.601529
Hepta‐2,5‐dienoylcarnitine	780.5478	45.61486	4.09691	0.000512	1.990032
3‐Dehydrosphinganine	134.7582	7.875713	4.09682	0.021055	1.467082
Pelubiprofen	990.5789	63.80088	3.95662	0.023486	1.549829
alpha‐Amylcinnamyl isovalerate	604.9535	42.86905	3.81882	0.019354	1.494239

**Table 9:** Metabolic molecules up‐regulated at higher expression levels in CD8^+^ T cell‐deficient mice than in normal mice following CD8^+^ T cell reflow.

name	Mean (C)	Mean(ZG)	log2FC	*p* value	VIP
3′‐Hydroxy‐HT2 toxin	9.750754963	257.173625	4.721085	0.015077	1.521496
Ferene triazine	6.684009357	151.940208	4.506646	0.033968	1.359613
ADP‐d‐ribose	88.2376219	982.848019	3.477503	0.00194	1.767394
Norcapsaicin	55.78469904	608.437564	3.447168	0.00098	1.809223
Daprodustat	24.25053613	201.709278	3.056189	0.045102	1.382979
Dapdiamide C	238.9035318	1958.44592	3.035209	0.037046	1.286246
(8S,9S,10R,13R,14S,17R)−5,6‐Dihydroperoxy‐10,13‐dimethyl‐17‐[(2R)−6‐methylheptan‐2‐yl]−1,2,3,4,6,7,8,9,11,12,14,15,16,17‐tetradecahydrocyclopenta[a]phenanthren‐3‐ol	245.94204	1919.75599	2.964533	0.001689	1.773084
PI(18:3(6Z,9Z,12Z)/PGJ2)	44.10285454	341.505361	2.952964	0.003151	1.721257
N‐Nervonoyl Aspartic acid	121.9643576	932.846144	2.93518	0.030786	1.349025
PA(10:0/20:4(6E,8Z,11Z,14Z)‐OH(5S))	69.38813877	529.869389	2.932876	4.85E‐05	1.872649
Tri‐N‐acetylchitotriose	59.84530085	443.534488	2.889736	0.028064	1.360836
3,7‐Dimethyl‐5‐octene‐1,7‐diol 1‐glucoside	12.5615447	92.320609	2.877639	0.025912	1.342773
OPC8‐CoA	64.55664025	471.581239	2.868869	0.030027	1.342597
PIP(PGF1alpha/18:0)	16.0037723	111.736487	2.803616	0.047323	1.233071
Ac‐Ser‐Asp‐Lys‐Pro‐OH	69.18998187	473.394223	2.774407	0.000739	1.844506

**Table 10:** Metabolic molecules down‐regulated at expression levels in CD8^+^ T‐cell‐deficient mice compared to normal mice after back‐infusion of CD8^+^ T cells.

name	Mean(C)	Mean(ZG)	log2FC	*p* value	VIP
3‐Dehydrosphinganine	134.7582	1.401845	−6.5869	0.017713	1.540732
Rokitamycin	134514.8	2616.685	−5.68388	0.015926	1.526097
Cer(d18:1/16:0)	127687.5	3185.666	−5.32488	0.017699	1.48933
Virol A	248.6604	10.21747	−4.60507	0.02883	1.448844
14,15‐DHET	476.6829	22.02382	−4.43589	0.02323	1.492314
alpha‐Amylcinnamyl isovalerate	604.9535	34.11167	−4.14849	0.018223	1.53016
Azaspiracid 4	65.01235	4.020113	−4.01541	0.035052	1.319837
Dioscoretine	183.0604	13.3042	−3.78237	0.024767	1.429458
(+)‐Vulgraon B	204.631	16.02513	−3.67462	0.01517	1.562538
Disofenin	184.9928	15.65949	−3.56236	0.038182	1.270733
Oblongolide	2324.386	199.7671	−3.54046	0.016803	1.545002
1,8‐Heptadecadiene‐4,6‐diyne‐3,10‐diol	548.8678	49.43843	−3.47275	0.019138	1.52087
Ursodeoxycholic acid	98803.73	9754.51	−3.34042	0.014539	1.56688
Chinenoside III	600.9274	59.70443	−3.33128	0.042899	1.355938
Arginine‐betaxanthin	98893.74	10415.41	−3.24716	0.012564	1.556281

### 3.7 Multivariate Statistical Analysis of Metabolites

PCA scatter plots were used for initial visualization of the complete dataset. To identify differential changes in hepatic metabolites induced by CD8^+^ T cell deficiency in mice, principal component analysis (PCA) was performed on liver metabolites using SIMCA. To better display metabolic variations between groups, orthogonal partial least squares discriminant analysis (OPLS‐DA) was performed on differentially grouped metabolites, as shown in Figures 6 and 7a,b,c. Figures 6 and 7 reveal distinct separation in the plots between the control group and the CD8^+^ T cell‐deficient group, as well as between the CD8^+^ T cell‐deficient group and the CD8^+^ T cell‐reinfused group. This indicates that CD8^+^ T cell depletion and subsequent reinfusion induced significant alterations in hepatic metabolic responses in mice. All samples fell within the 95% confidence ellipse. To avoid potential overfitting during model construction in OPLS‐DA analysis, the model validity was verified using a permutation test (Figure 7b,d).

**Figure 6:**
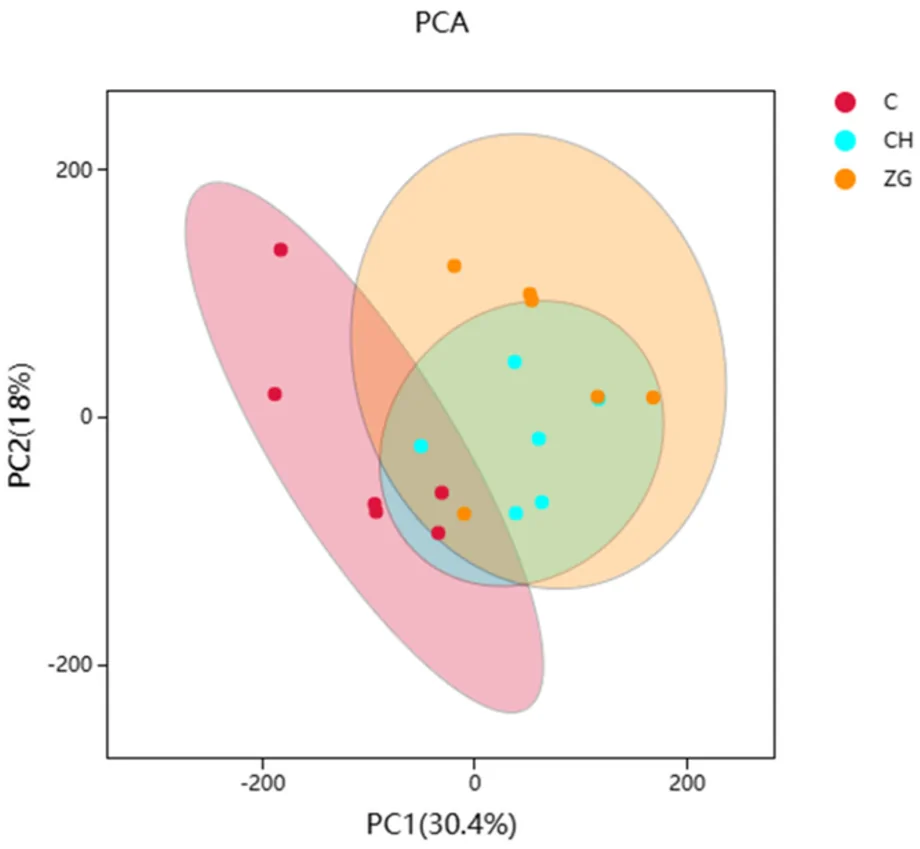
Graph of PCA analysis. The PCA score graph utilizes the scores of the first and second principal components (PC1 and PC2) as the coordinate axes, illustrating the group attribution of the samples through scattered points of varying shapes.

**Figure 7:**
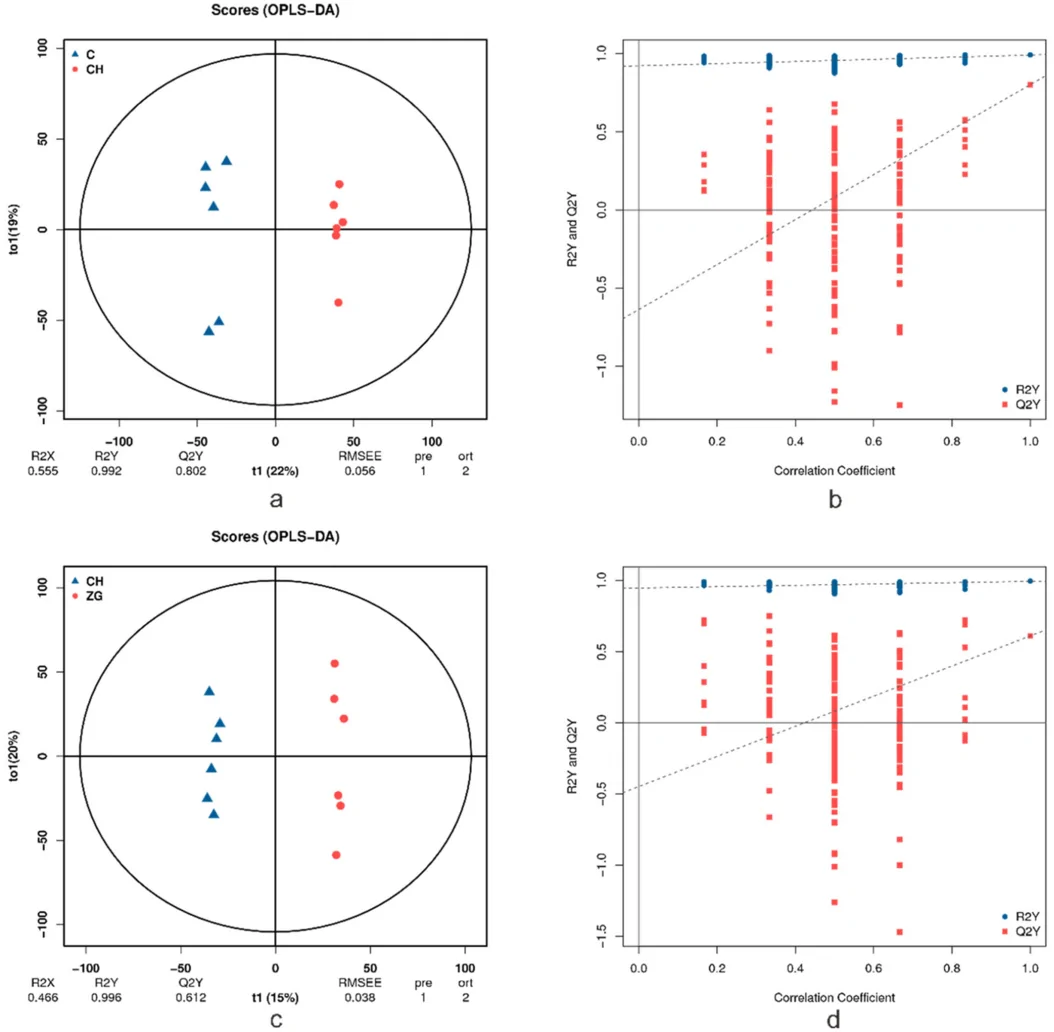
OPLS‐DA analysis diagram. Figures (a) and (c) display the OPLS‐DA scoring outcomes, illustrating sample distribution according to the predicted principal component (t1) and the orthogonal principal component (to1), with group differentiation denoted by scatter shapes. The permutation test graphs (b, d) demonstrate a correlation between permutation retention and the values of Q2 and R2Y, affirming the model's consistent predictive and explanatory performance.

### 3.8 Hierarchical Cluster Analysis of Differential Metabolites

We identified differentially expressed metabolic molecules meeting the criteria of VIP > 1, *p* < 0.05, and |log2FC|> 2. As shown in Figure 8a, in comparison to the normal control group, the CD8^+^ T cell defect group exhibited increased expression levels of 3alpha,7alpha,12alpha‐trihydroxy‐5beta‐cholestane, 1‐(6Z,9Z,12Z‐octadecatrienoyl)‐glycero‐3‐phosphate, Cer(d20:1/20:3(5Z,8Z,11Z)‐O(14R,15S)), 7‐Deaza‐2′‐deoxyguanosine, 4‐Hydroxynonenal, and 7‐Hydroxyoctadecanoic acid, while the expression of 4‐methyl‐2‐oxopentanoate decreased. Specifically, 7‐Hydroxyoctadecanoic acid is associated with α‐linolenic acid metabolism, and 1‐(6Z,9Z,12Z‐octadecatrienoyl)‐glycero‐3‐phosphate serves as a precursor for arachidonic acid synthesis. 4‐Hydroxynonenal is a characteristic end product of arachidonic acid metabolism. 7‐Deaza‐2′‐deoxyguanosine is synthesized by Catellatospora, and Cer(d20:1/20:3(5Z,8Z,11Z)‐O(14R,15S)) is an end product of sphingolipid metabolism highly correlated with it. Conversely, in the CD8^+^ T cell defect group, the expression levels of 4‐Methyl‐2‐oxopentanoate, 5‐Aminopentanoate, N‐Acetoxyethylflindersine, Hepta‐2,5‐dienoylcarnitine, trans‐2‐Dodecenoylcarnitine, 14,15‐DHet, and alpha‐Amylinoamyl isovalerate significantly decreased. In contrast, as shown in Figure 8b, compared to the normal control group, the CD8^+^ T cell reinfusion group showed decreased expression levels of Ursodeoxycholic acid, Oblonglide, (+)‐Vulgraon B, and 1,8‐Heptadecadiene‐4,6‐diyne‐3,10‐diol, while the expression of alpha‐Amylcinnamyl isovalerate decreased. Conversely, the expression levels of Ferene triazine, Dapdiamide C, 3′‐Hydroxy‐HT2 toxin, ADP‐d‐ribose, and Daprodustat increased in the CD8^+^ T cell reinfusion group compared to the normal control group.

**Figure 8:**
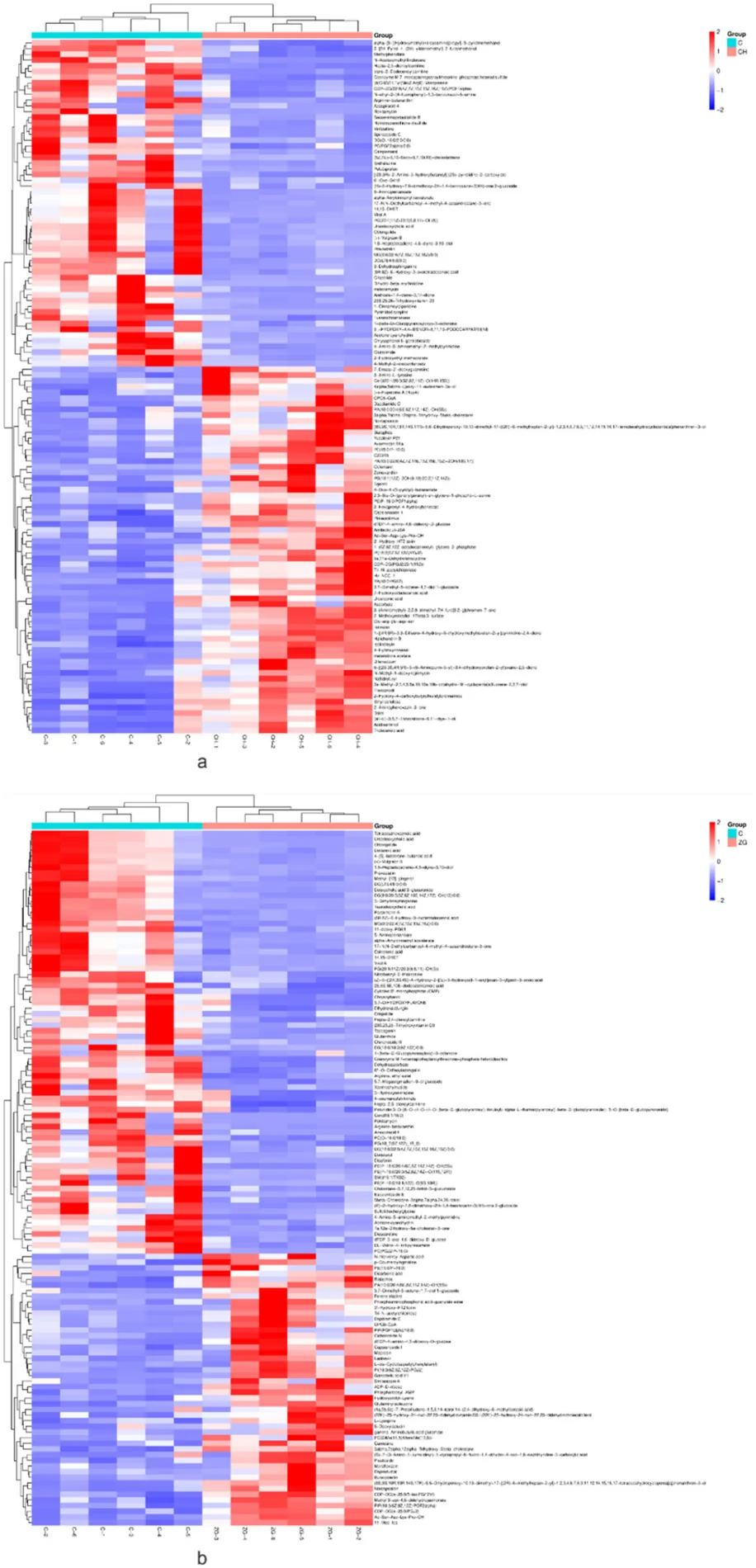
Heat map of differential metabolite clustering. (a) Comparison of normal control and CD8^+^ T cell‐deficient groups; (b) Comparison of normal control and CD8^+^ T cell‐returned groups.

### 3.9 Differential Metabolite KEGG Enrichment Analysis

As shown in Figure 9a,b, the results of KEGG enrichment revealed that CD8^+^ T‐cell‐deficient mice and normal control mice differed in metabolic molecules involved in several pathways, including biosynthesis of valine, leucine, and isoleucine, metabolism of α‐linolenic acid, metabolism of sphingolipids, degradation of valine, leucine, and isoleucine, biosynthesis of steroids, metabolism of arachidonic acid, biosynthesis of protic bile acids synthesis, and xenobiotic metabolism of cytochrome P450 metabolism. Of these, the biosynthesis of valine, leucine, and isoleucine was significantly associated with CD8^+^ T cell defects. As shown in Figure 9c,d, the differences in metabolic molecules in CD8^+^ T‐cell‐deficient mice compared with normal controls after the infusion of CD8^+^ T‐cells back to the CD8^+^ T‐cell‐deficient mice involved the pathways of α‐linolenic acid metabolism, histidine metabolism β‐alanine metabolism, sphingolipids metabolism, arachidonic acid metabolism, native bile acids biosynthesis, fatty acid biosynthesis and purine metabolism.

**Figure 9:**
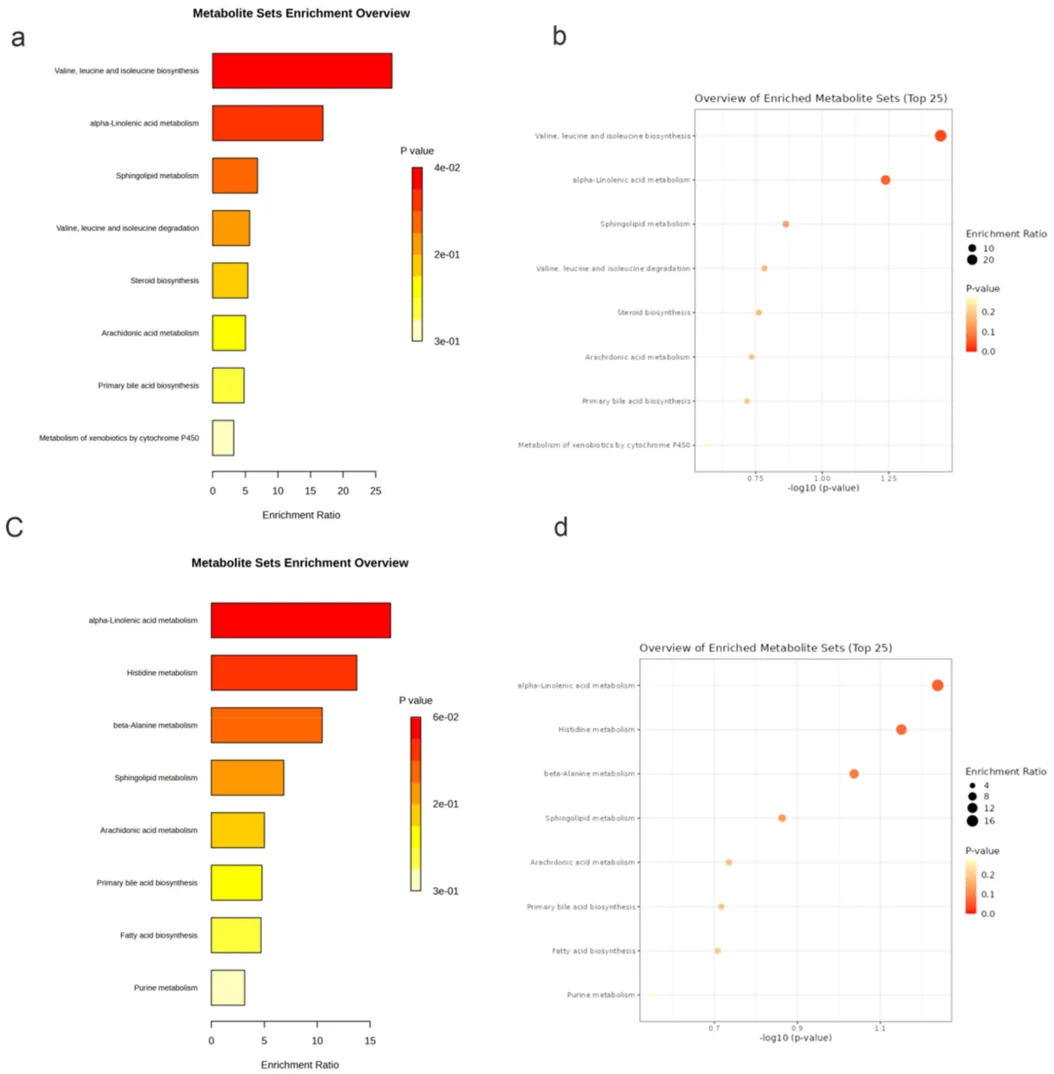
KEGG enrichment analysis of differential metabolites. *Note:* (a and b) are bar and bubble plots comparing the normal control and CD8^+^ T‐cell‐deficient groups, respectively; and (c and d) are bar and bubble plots comparing the normal control and CD8^+^ T‐cell‐deficient groups, respectively.

### 3.10 Microbiota‐Metabolite Association Analysis

At the genus level, we performed an integrative analysis of gut microbiota diversity combined with untargeted metabolomics. As shown in Figure 10a,c,e, compared with the control group, the relative abundances of *unclassified_Clostridia_UCG_014*, *Intestinimonas*, *uncultured_Lachnospiraceae_bacterium*, and *Family_XIII_AD3011_group* in the gut were significantly elevated in CD8^+^ T cell‐deficient mice (*p* < 0.05). In the liver, the levels of PE(P‐18:0/PGF1alpha), 2,3‐Bis‐O‐(geranylgeranyl)‐sn‐glycero‐1‐phospho‐l‐serine, Capsianoside II, Naftidrofuryl, and 6‐[(2S,3S,4R,5R)−5‐(6‐aminopurin‐9‐yl)−3,4‐dihydroxyoxolan‐2‐yl]oxane‐2,5‐dione were upregulated. Conversely, the hepatic levels of trans‐2‐Dodecenoylcarnitine, Vinfosiltine, CDP‐DG(22:6(4Z,7Z,10Z,13Z,16Z,19Z)/PGF1alpha), and Hepta‐2,5‐dienoylcarnitine were downregulated. Notably, *Family_XIII_AD3011_group* was significantly and inversely correlated with trans‐2‐dodecenoylcarnitine and hept‐2,5‐dienoylcarnitine.

**Figure 10:**
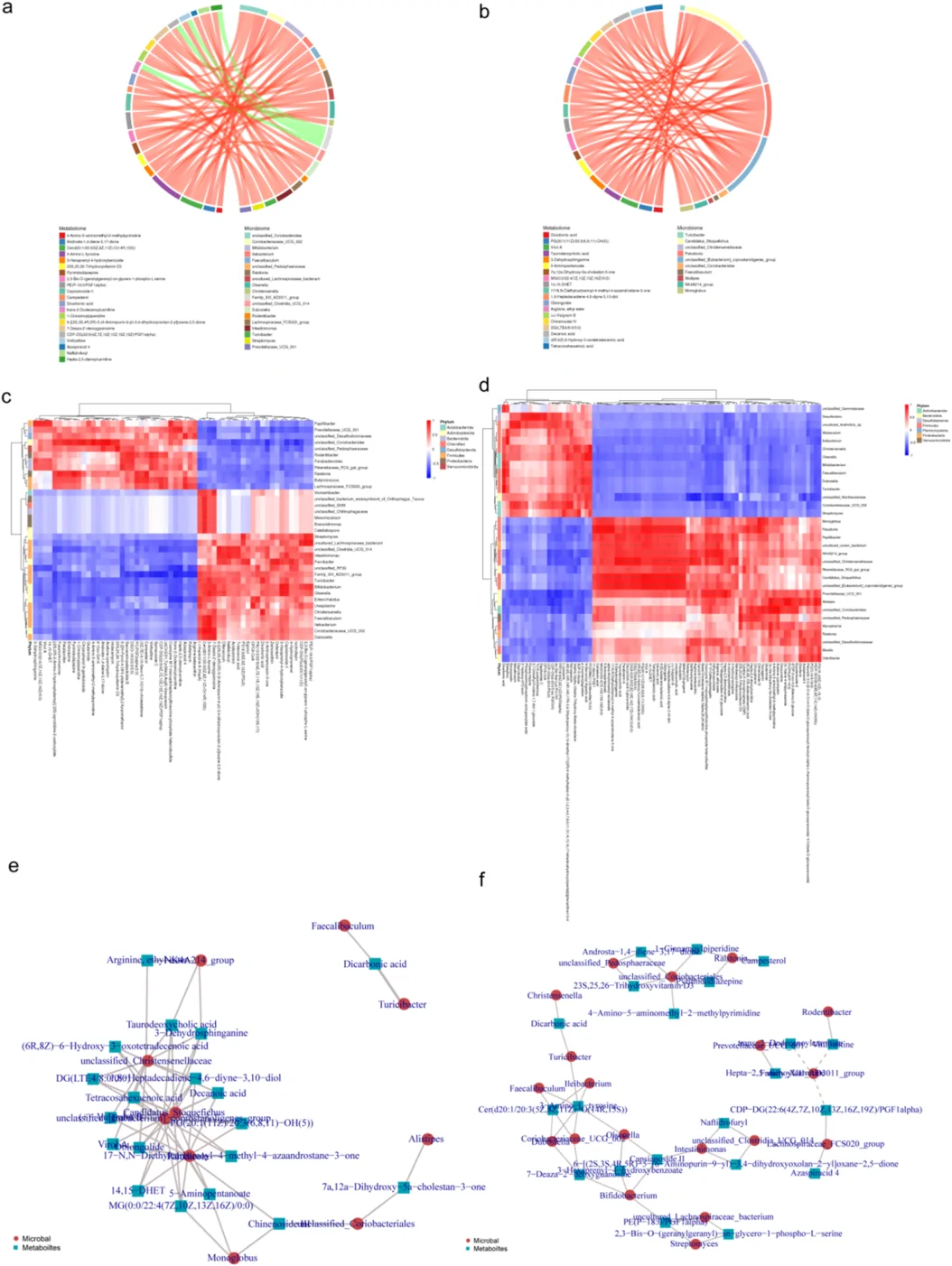
Microbiota‐metabolite association analysis. *Note:* (a and b) Chord diagrams showing correlations between differential genera and differential metabolites. The left side represents the metabolome and the right side represents the microbiome; (c and d) Spearman's correlation heatmaps between differential genera and differential metabolites. Rows represent genera and columns represent metabolites; (e and f) Microbiota–metabolite correlation networks. Red circular nodes represent microbial genera, and cyan square nodes represent metabolites; solid lines indicate positive correlations and dashed lines indicate negative correlations. (a, c, e) Comparison between the control group and the CD8^+^ T cell‐deficient group; (b, d, f) comparison between the control group and the CD8^+^ T cell reconstitution group.

## 4 Discussion

CD8^+^ T cells play a crucial role in the immune system by identifying and eliminating infectious pathogens or abnormal cells [4, 5, 7, 15, 16, 17, 18]. CD8^+^ T cells, present predominantly in lymphoid organs and abundant in the intestinal epithelium, play a crucial role in the immune system by releasing perforin and granzyme to induce target cell death via the Fas‐FasL pathway. These cells collaborate with intestinal epithelial cells to support beneficial microorganism colonization, immune barrier formation, and antigen tolerance. The impact of CD8^+^ T cell deficiency or dysfunction on intestinal microbiota composition and liver metabolism mechanisms requires further elucidation. The gut microbiota, comprising myriad microorganisms, significantly contributes to immune homeostasis, metabolic processes, and host defense against pathogens, influencing CD8^+^ T cell activation and function diversely [19, 20, 21, 22, 23, 24, 25, 26, 27, 28]. Metabolites generated by gut microbes can impact CD8^+^ T cell function, with butyrate, for instance, directly influencing CD8^+^ T cell activity to enhance anti‐tumor immune responses and robustly stimulate IFN‐γ secretion by CD8^+^ T cells [29, 30]. This offers a novel avenue for modulating CD8^+^ T cell function in anti‐tumor immunity and pathogen resistance.

Dysbiosis of the gut microbiota, encompassing both structural and functional perturbations, is closely associated with multiple immune cell lineages, including dendritic cells, macrophages, CD4^+^ T cells, CD8^+^ T cells, and B cells [31]. Accordingly, in the present study, we employed CD8^+^ T cell‐deficient mice as the experimental model and adopted a strategy of repeated adoptive transfer of CD8^+^ T cells every 3 days to sustain physiological CD8^+^ T cell levels in vivo. By integrating gut microbiota sequencing with hepatic untargeted metabolomics, we systematically investigated how CD8^+^ T cell abundance shapes the gut microbial community structure and liver metabolism. In this study, adoptive transfer of CD8^+^ T cells into CD8^+^ T cell‐deficient mice led to a reduction in gut microbial alpha diversity and a decline in species abundance. Beta diversity analysis further revealed that the overall microbial community structure differed significantly between the normal control group and the CD8^+^ T cell‐deficient group. These findings suggest that certain bacterial taxa undergo unchecked overgrowth in the absence of intestinal CD8^+^ T cell‐mediated immune surveillance, and that this dysbiotic expansion can be reversed upon restoration of CD8^+^ T cell populations via adoptive transfer. These observations collectively support the notion that the intestinal immune system and the gut microbiota engage in reciprocal regulation to jointly maintain gut microbial homeostasis [32].

Significant alterations in microbial diversity were observed in this study, particularly evident at the family and genus levels alongside CD8^+^ T cell deficiencies. Bacterial abundances of *unclassified_Clostridia_UCG_014*, *unclassified_RF39*, *Intestinimonas*, and *unclassified_RF39* exhibited an increasing trend, which reversed after the reinfusion of CD8^+^ T cells. This reversal suggests a correlation between the fluctuation in these fungi's abundance and CD8^+^ T cell levels. *Intestinimonas*, a probiotic from the *firmicutes* phylum found in mice intestines, facilitates the conversion of dietary fructose and lysine into butyrate [33, 34, 35]. Following CD8^+^ T cell depletion in this study, the levels of *Intestinimonas* and *unclassified_RF39* bacteria in the mice intestines rose. Butyrate, essential for intestinal epithelial cell energy and crucial for human colon health maintenance [16, 19, 36, 37, 38, 39, 40], possesses anti‐inflammatory properties [16, 33, 35, 38, 41, 42, 43, 44]. Butyrate can augment bacterial clearance by macrophages, preserve gut microbiota balance, and homeostasis by up‐regulating Host Defense Peptide expression [40, 45, 46]. Research indicates that butyrate modulates CD8^+^ T cells by suppressing Histone Deacetylase activity [29, 43, 47]. *Intestinimonas* may contribute to maintaining gut microbiota diversity and homeostasis via butyrate when CD8^+^ T cells are deficient. Integrated microbiome and untargeted metabolomics analysis revealed that trans‐2‐dodecenoylcarnitine and hepta‐2,5‐dienoylcarnitine were significantly and inversely correlated with the abundance of *Family_XIII_AD3011_group*. Both metabolites are trans‐2‐enoylcarnitines, established intermediates of mitochondrial β‐oxidation, and their reduced abundance suggests a decline in hepatic mitochondrial fatty acid oxidation flux. This reduction may be attributed to diminished fatty acid demand following CD8^+^ T cell deficiency.

Cellular metabolism directly influences the function and immune response of CD8^+^ T cells through the regulation of pathways such as energy production, signal transduction, and biosynthesis. CD8^+^ T cells must rapidly enhance ATP production to fulfill their energy requirements when transitioning from a quiescent state to an activated state for effector functions. Research indicates that activated CD8^+^ T cells enhance ATP synthesis by promoting glycolytic pathways and mitochondrial oxidative phosphorylation, thereby supporting cell proliferation and effector functions [1, 5, 17, 48]. During the period in which CD8^+^ T cells exert their effects, efficient energy metabolism is essential for maintaining their functions. Studies demonstrate that CD8^+^ T cells primarily rely on fatty acid oxidation as their main energy source during effector phases and must regulate intracellular nutritional sensors and metabolic pathways to preserve functional stability [5, 49]. Moreover, recent investigations have highlighted the critical role of CD8^+^ T cells in the anti‐tumor immune response and the importance of cellular metabolism in modulating tumor immune escape. Consequently, a comprehensive understanding of the metabolic regulatory mechanisms governing CD8^+^ T cells not only elucidates the nature of immune responses but also provides a theoretical foundation for the development of innovative immunotherapy strategies. In CD8^+^ T cell‐deficient mice, alterations were observed in the biosynthesis of valine, leucine, and isoleucine, alpha‐linolenic acid metabolism, sphingolipid metabolism, degradation of valine, leucine, and isoleucine, and biosynthesis of steroids compared to normal mice. Upon reinfusion of isolated CD8^+^ T cells into CD8^+^ T cell‐deficient mice, differences persisted in alpha‐linolenic acid metabolism, histidine metabolism, β‐alanine metabolism, sphingolipid metabolism and arachidonic acid metabolism compared to normal mice. A study investigating the impact of CD8^+^ T cell deficiency on liver metabolism in mice compared control group mice with CD8^+^ T cell‐deficient mice. Enrichment analysis of 121 differentially metabolized molecules revealed significant enrichment in the biosynthesis pathways of valine, leucine, and isoleucine, suggesting that the influence of CD8^+^ T cell deficiency on liver metabolism primarily manifests in the biosynthesis pathway of these amino acids. Valine, leucine, and isoleucine are branched‐chain amino acids that play a crucial role in regulating energy homeostasis, nutritional metabolism, intestinal health, immunity, and disease in humans and animals [50].

When CD8^+^ T cells recognize antigens via their T cell receptors (TCR) and become activated, they undergo rapid proliferation and differentiation, which necessitates a substantial supply of proteins. Branched‐chain amino acids, particularly leucine, are crucial not only for protein synthesis but also for alleviating the inhibition of mTORC1 through sensor proteins. This process promotes the proliferation and differentiation of CD8^+^ T cells into effector T cells [51, 52]. In this study, the deficiency of CD8^+^ T cells significantly disrupted the metabolic pathway of branched‐chain amino acids. Notably, 4‐methyl‐2‐oxopentanoate, a degradation product of leucine, was expressed at significantly lower levels in the CD8^+^ T cell deficiency group, indicating that branched‐chain amino acids were in a highly expressed state. The mTOR signaling pathway is regulated by the body's feedback mechanisms to sustain intestinal immune function. The mTOR signaling pathway significantly influences the function and development of CD8^+^ T cells, thereby regulating their role in immune responses [53, 54]. Inadequate intake of branched‐chain amino acids may result in lymphocyte depletion, increasing susceptibility to pathogens. Furthermore, branched‐chain amino acids can boost SIgA secretion, thereby enhancing the defense capability of the intestinal mucosal surface, inhibiting pathogen invasion into the lamina propria, and enhancing host immunity [55]. These amino acids can modulate immune function by augmenting the energy supply for immune cells, CD4^+^ T cells, CD4^+^ T cells/CD8^+^ T cells, intestinal immunoglobulins, innate and adaptive immune responses, pro‐inflammatory cytokines, and dendritic cell functions. Our experimental findings suggest a close association between the metabolic processing of branched‐chain amino acids in the liver and CD8^+^ T cell dysfunction. Furthermore, branched‐chain amino acids may play a significant role in modulating immune responses mediated by CD8^+^ T cells. Discrepancies in the abundance of intestinal microbiota appear to be linked to variations in CD8^+^ T cell populations. The intestinal microbiota likely impacts CD8^+^ T cell numbers by modulating butyrate levels in the gut and influencing the concentrations of specific factors like IL‐10 in the bloodstream, thereby enhancing their efficacy in combating infections and tumor development.

Several limitations of the present study should be acknowledged. First, we employed a systemic *Cd8a* knockout model in which CD8^+^ T cells were reconstituted in a non‐subset‐specific manner; consequently, the observed alterations in the gut microbiota and hepatic metabolism cannot be unambiguously attributed to the loss of any particular CD8^+^ T cell subset. Second, although we adoptively transferred spleen‐derived CD8^+^ T cells into CD8^+^ T cell‐deficient mice via the tail vein, we did not characterize the proportional representation or absolute numbers of tissue‐resident memory CD8^+^ T (CD8^+^ TRM) cells in the intestinal mucosa or the liver, nor did we directly assess intestinal permeability or serum levels of lipopolysaccharide (LPS), a marker of bacterial translocation. Accumulating evidence indicates that distinct CD8^+^ T cell subsets—particularly tissue‐resident memory T cells—play highly specialized roles in local immunity within the gut and liver [32, 56, 57, 58]. Therefore, whether the observed gut microbiota and hepatic metabolic changes are driven by the global CD8^+^ T cell pool or by specific intestinal and hepatic CD8^+^ T cell subsets remains to be fully elucidated. Future studies employing subset‐specific adoptive transfer of CD8^+^ TRM cells, combined with functional assessment of intestinal barrier integrity, will be required to establish a mechanistic link between intestinal CD8^+^ T cells and the gut–liver axis. The present study revealed that hepatic metabolic pathways were altered in CD8^+^ T cell‐deficient mice, particularly the biosynthesis of valine, leucine, and isoleucine. However, the mechanisms by which CD8^+^ T cells regulate these pathways—or how these metabolic alterations in turn affect CD8^+^ T cell function—remain to be fully elucidated. Furthermore, the present study did not interrogate the status of the mTOR signaling pathway following CD8^+^ T cell deficiency; these aspects warrant in‐depth investigation in future studies. In addition, exogenously transferred CD8^+^ T cells administered via the tail vein exhibited limited in vivo persistence, requiring repeated supplementation every 3 days to sustain a stable circulating CD8^+^ T cell pool—a reconstitution regimen that deviates substantially from physiological steady‐state conditions. The relatively small sample size of the present study warrants cautious interpretation; future large‐scale replication studies are needed to validate these findings and reduce potential statistical bias. Moreover, more refined use of the existing *Cd8a* knockout model—including the incorporation of well‐matched control groups and the extension of this approach into disease‐relevant settings—will be essential to dissect the specific contribution of CD8^+^ T cells to gut microbial regulation. Despite these caveats, the present study provides an initial foundation for future investigations aimed at elucidating how CD8^+^ T cells shape gut and hepatic metabolic homeostasis. Finally, all experiments in this study were conducted using female mice, as male mice are prone to aggressive fighting behavior. Sex is a well‐established biological variable that influences gut microbial composition, immune function, and hepatic metabolism. Subsequent studies in male mice are planned to determine whether sex‐related differences modify the observed phenotypes [31, 59].

## Author Contributions

**Zhongshi Dai:** funding acquisition, data curation, supervision, resources, writing – review and editing, project administration. **Chuan Wang:** Project administration, software. **Hu Mu:** project administration. **Chunxiu Hou:** conceptualization, investigation, methodology, and software. **Jing Tang:** writing – review and editing. **Jinhai Ma:** supervision, writing – review and editing, methodology, formal analysis, project administration. **Bingshuo Qian:** methodology, validation, visualization, formal analysis, software, investigation, and conceptualization. **Mingxing Zhu:** funding acquisition, data curation, supervision, resources, writing – review and editing, project administration.

## Ethics Statement

This study and the experimental procedures included have been approved by the Medical Research Ethics Review Committee of the General Hospital of Ningxia Medical University (Ethical Approval No.: KYLL‐2023‐0461), and the approval letter from the Ethics Review Committee has been provided. Moreover, all animal housing and experimental procedures were conducted in strict accordance with the institutional guidelines for the care and use of laboratory animals.

## Conflicts of Interest

The authors declare no conflicts of interest.

## Data Availability

The data related to the results of this study are included in this paper and its attached files. The datasets presented in this study can be found in online repositories. The name of the repository and the accession number(s) can be found below: NCBI, accession number PRJNA1453230.
